# Evaluating the effects of antidepressant medication on post-operative outcomes in lower body contouring surgery after massive weight loss: A retrospective study

**DOI:** 10.1016/j.jpra.2025.09.018

**Published:** 2025-09-21

**Authors:** Susanna Pajula, Esko Veräjänkorva, Virve Koljonen, Max Karukivi

**Affiliations:** a Department of Plastic and General Surgery, Turku University Hospital, Kiinamyllynkatu 4-8, 20500 Turku, Finland; b Hospital Mehiläinen-NEO, Turku Finland; c Department of Plastic Surgery, University of Helsinki and Helsinki University Hospital, Helsinki, Finland; d Department of Adolescent Psychiatry, University of Turku and Turku University Hospital, Turku, Finland

**Keywords:** Massive weight loss, Lower body contouring surgery, Antidepressant medication, Postoperative complications

## Abstract

**Background:**

Postoperative complications are common following lower body contouring surgery (LBCS) after massive weight loss (MWL). This study aimed to determine whether antidepressant use is associated with the occurrence of complications after LBCS.

**Materials and Methods:**

This retrospective study included consecutive patients who underwent LBCS after MWL at Turku University Hospital in Finland between 2016 and 2021. Weight loss was achieved either through bariatric surgery (BS) or lifestyle modifications.

**Results:**

A total of 150 patients were included, with a mean age of 45.8 ± 11.1 years. The majority were women, 128 (85.3 %), and 22 (14.7 %) were men. Most patients (71.3 %) underwent BS. Antidepressant use was reported in 33 patients (22 %). Compared to non-users, antidepressant users were older (49.1 vs. 44.8 years, *p* = 0.05) and had a significantly higher body mass index (BMI) at the time of LBCS (29.5 ± 5.5 vs. 27.8 ± 3.0 kg/m², *p* = 0.02). The overall complication rate was 51.3 %. Patients on antidepressants had a 4.4-fold increased risk of developing deep tissue infection after surgery (aOR 4.36, 95 % CI: [1.08–17.71], *p* = 0.04) and were over five times more likely to be rehospitalized for intravenous antibiotic treatment (aOR 5.07, 95 % CI: [1.28–20.02], *p* = 0.02) than those who did not use antidepressants.

**Conclusion:**

Antidepressant use was associated with a higher risk of deep tissue infection. Whether these findings are due to the antidepressant or the underlying psychiatric condition remains unclear. Future studies should investigate the relative contributions of these factors to optimize the care of patients with a history of MWL who undergo LBCS.

## Introduction

Despite the numerous positive health and psychosocial effects of massive weight loss (MWL), it may lead to undesirable consequences. The most prominent of these is the development of excess skin folds, typically located around the lower abdomen, flanks, lower back, upper arms, breasts, and inner thighs.^1^

Excess skin may cause a range of physical, functional, and psychosocial issues.^2^^,^^3^ Psychosocial symptoms include shame, reduced self-esteem, avoidance of social situations, and even social isolation.^4^^,^^5^ As a result, although MWL initially improves quality of life (QoL), the presence of excess skin may eventually reduce QoL and contribute to the development of depressive symptoms.^6^^,^^7^ Up to 90 % of patients with MWL experience symptoms related to excess skin.^3^ Surgical body contouring is the only effective treatment. In Finland, public healthcare primarily offers lower body contouring surgery (LBCS) to address the functional and health-related impairments caused by excess skin.^8^

However, up to 50 % of patients undergoing LBCS after MWL experience postoperative complications to some degree.^9^^,^^10^ Risk factors such as preoperative weight, age, smoking, amount of tissue removed, and underlying health conditions are known to increase the risk of complications.^11^^,^^12^ Common complications include impaired wound-healing, excessive seroma secretion, and postoperative hematoma.^9^^,^^13^ Serious, life-threatening complications are extremely rare.^14^

Psychological health has been shown to affect postoperative wound healing.^15^^,^^16^ Stress, anxiety, and depression are associated with delayed wound healing, increased pain, and a higher risk of infections.^16^^,^^17^ Antidepressants are frequently prescribed to manage mental health disorders and to alleviate significant psychological distress. Therefore, in addition to patients’ mental health, the pharmacological treatment they receive may also contribute to the risk of complications. For example, selective serotonin reuptake inhibitors (SSRIs) have been linked to an increased risk of postoperative bleeding, although the evidence is somewhat conflicting.^18^^,^^19^ To our knowledge, no previous studies have examined the association between antidepressant use and postoperative complications in patients undergoing LBCS. Therefore, this single-center retrospective study aimed to assess the prevalence of antidepressant use among patients undergoing LBCS and to evaluate whether such use is associated with an increased risk of postoperative complications.

## Patients and methods

This retrospective chart review was conducted at the Department of Plastic and General Surgery at Turku University Hospital, a tertiary referral center in Finland. The study protocol was approved by the hospital’s Institutional Review Board (approval number VSSHP/2023/135829). As this was a retrospective chart review with no patient contact, informed consent was not required, according to Finnish legislation. The legal basis for processing personal data is public interest and scientific research (EU General Data Protection Regulation 2016/679 (GDPR), Article 9(2)(j); Data Protection Act, Sections 4 and 6). *The manuscript was checked against the Strengthening of the Reporting of Observational Studies in Epidemiology (STROBE) checklist.*

The hospital’s electronic database, Opera®, was queried to identify patients who underwent LBCS between 1 January 2016 and 31 December 2021. The LBCS procedures included traditional abdominoplasty, body lift/belt lipectomy, and panniculectomy. Eligibility criteria for LBCS included a history of clinically defined MWL without a strict numerical threshold and the presence of health-related and functional symptoms caused by an excess skin fold in the lower body region. In addition, patients were required to have a body mass index (BMI) below 30 kg/m² and a stable weight prior to surgery. Stable weight was defined as the self-reported achievement of a target weight with ongoing weight loss before LBCS*.* A higher BMI threshold was accepted for panniculectomy procedures. Purely aesthetic concerns were not considered sufficient for surgery in the public health-care system. Patients who had undergone post-bariatric body contouring procedures on other anatomical regions such as the upper arms, inner thighs, or breast were excluded. Similarly, patients who underwent LBCS for indications such as post-pregnancy changes or rectus diastasis were excluded as these cases were not related to MWL.

A detailed review of electronic medical records was conducted to collect data on age, gender, comorbidities (hypertension and diabetes mellitus), smoking status, highest lifetime weight, BMI, and method of weight loss (BS or non-surgical method). The LBCS-specific variables included current weight and BMI at the time of surgery. In addition, the type of LBCS procedure, length of hospital stays (in days), and all postoperative complications were recorded. Complications were categorized both by timing- immediate (within 24 h), delayed (1–30 days), and late (more than 30 days postoperatively)—and by severity, according to the Clavien-Dindo classification system.^20^

Data on antidepressant medication was collected from the National Pharmacy Database and the electronic medical records. Antidepressant use was analyzed both in terms of overall use and by pharmacological subtype. SSRIs and serotonin-norepinephrine reuptake inhibitors (SNRIs) were grouped together as the SSRI/SNRI group, while tricyclic antidepressants and other types were classified as the other antidepressant group. In this study, antidepressant medications used prior to or initiated after the LBCS procedure were not considered.

The primary exposure variable was defined as the use of antidepressants at the time of LBCS, based on national prescription data and electronic medical records. Patients with an active prescription on the day of surgery were classified as users. The primary outcome was the occurrence of any postoperative complication both within and beyond 30 days. Secondary outcomes included the type and severity of complications (according to the Clavien-Dindo classification), reoperation, and delayed initiation of intravenous antibiotics.

## Statistical methods and analysis

Statistical analyses were performed using SPSS® Statistics version 29.0 (IBM® Corporation, NY, USA) and GraphPad Prism version 10.4 (GraphPad Software Inc., CA, USA). The D’Agostino–Pearson normality test was used to assess the normality of the variables. Continuous variables with parametric distributions were described using means with standard deviations (SD), whereas non-parametric variables were summarized as medians with interquartile ranges (IQR). Categorical variables were compared between antidepressant users and non-users using the chi-squared test or Fisher’s exact test, while continuous variables were compared using the Student’s *t*-test or the Mann–Whitney *U* test, as appropriate. Risk factors for complications were analyzed using Fisher’s exact test. Multivariable logistic regression was used to examine the association between antidepressant use and postoperative outcomes, including any complication and treatment-related complications. The models were adjusted for age, BMI at the time of surgery, diabetes, hypertension, smoking status, and length of hospital stay. Results are reported as adjusted odds ratios (aOR) with 95 % confidence intervals (CIs). Statistical significance was set at *p* < 0.05.

## Results

The specific inclusion criteria resulted in the selection of 150 patients with a mean age of 45.8 ± 11.1 years (range: 21–74 years). The majority of patients were women, 128 (85.3 %), with a mean age of 46.1 ± 10.5 years, while only 22 (14.7 %) were men, with a mean age of 44.1 ± 14.3 years (*p* = 0.27). In total, 45 patients (30.0 %) had hypertension, and 25 (16.7 %) had type 2 diabetes mellitus as a somatic comorbidity. Table 1 summarizes the patients' demographic characteristics.

**Table 1:** Demographic characteristics of 150 patients who underwent lower body contouring surgery (LBCS) during 2016–2021 after MWL. The *p*-value reflects the probability of the observed difference between two groups regarding the use of any antidepressant medication.

	All patients^a^	Antidepressant-group	Non Antidepressant-group	*p*-value^c^
*n* (%)	150 (100)	33 (22)	117 (78)	
Age	45.8 ± 11.1	49.1 ± 10.1	44.8 ± 11.2	0.05
Women	128 (85.3)	31 (93.9)	97 (82.9)	0.16
Men	22 (14.7)	2 (6.1)	20 (17.1)	
**Smoking status**				0.48
No smoker	118 (78.7)	28 (84.9)	90 (76.9)	
Former smoker	14 (9.3)	3 (9.1)	11 (9.4)	
Current smoker	18 (12.0)	2 (6.1)	16 (13.7)	
Highest lifetime weight (kg)	125.8 ± 21.5	126.8 ± 22.6	125.5 ± 21.3	0.76
Highest lifetime BMI (kg/m^2^)	45.0 ± 5.9	45.7 ± 7.4	44.7 ± 5.4	0.41
**Weight loss method**				0.05
Bariatric surgery	107 (71.3)	28 (84.9)	79 (67.5)	
Non-surgery method	43 (28.7)	5 (15.1)	38 (32.5)	
Total amount of weight loss (kg)	47.0 ± 15.8	45.2 ± 15.8	47.5 ± 15.8	0.45
BMI at time of LBCS	28.1 ± 3.7	29.5 ± 5.2	27.8 ± 3.0	0.02^b^
**LBCS**				
Abdominoplasty (Traditional)	128 (85.3)	29 (87.9)	99 (84.6)	0.79
Bodylift/ Belt lipectomy	12 (8.0)	0 (0)	12 (10.3)	0.07
Panniculectomy	10 (6.7)	4 (12.1)	6 (5.1)	0.23
Removed tissue (g) (median, IQR)	1728.8 (1263.5–2368.5)	2030 (1243–2500)	1721 (1277–2360)	0.62
Hospital stay after LBSC (days)	3.7 ± 1.24	3.3 ± 0.98	3.8 ± 1.3	0.03^b^

a Parametric values are reported as mean ± SD, except for the number of patients marked as *n* (%).

b Indicates a statistically significant finding. Statistical significance was set at *p* < 0.05.

c Chi-square test.

### Massive weight loss and body contouring surgery

Altogether, 107 patients (71.3 %) had undergone BS, while 43 (28.7 %) had achieved weight loss through non-surgical methods. Most BS (78.5 %) were performed at Turku University Hospital, while the remaining procedures were conducted in another hospital district (13.1 %), a private hospital (7.5 %), or abroad (1.0 %). The mean total weight loss was 47.0 ± 15.8 kg Table 1.

At the time of LBCS, the patients' mean weight was 78.8 ± 12.7 kg, and their mean BMI was 28.1 ± 3.7 kg/m^2^. Altogether, 128 patients (85.3 %) underwent traditional abdominoplasty, 12 (8 %) underwent a body lift, and 10 (6.7 %) underwent panniculectomy as their lower body contouring procedure. Liposuction was performed concurrently with LBCS in 39 patients (26.0 %) as a combined procedure. The median weight of resected tissue was 1728.8 g (IQR: 1263.5–2368.5), with a range from 506.0 to 7600.0 g. All patients received a perioperative dose of antibiotics at the time of anesthetic induction, in accordance with institutional guidelines. These guidelines account for factors such as potential antibiotic allergies and surgical site contamination risk. The majority of patients, 131 (87.3 %), received a second-generation cephalosporin, specifically cefuroxime, at a dose of 1.5 g. In cases where cephalosporin was contraindicated, 16 patients (10.7 %) received a lincosamide (clindamycin 600 mg), two (1.3 %) received a fluoroquinolone (levofloxacin 500 mg), and one (0.7 %) received a carbapenem (meropenem 1 g). Postoperative antibiotics were prescribed to 110 patients (73.3 %). The mean duration of hospital stay following LBCS was 3.7 ± 1.24 days (range: 1–9 days). None of the patients required intensive care following surgery.

### Antidepressant medication

**Figure 1:**
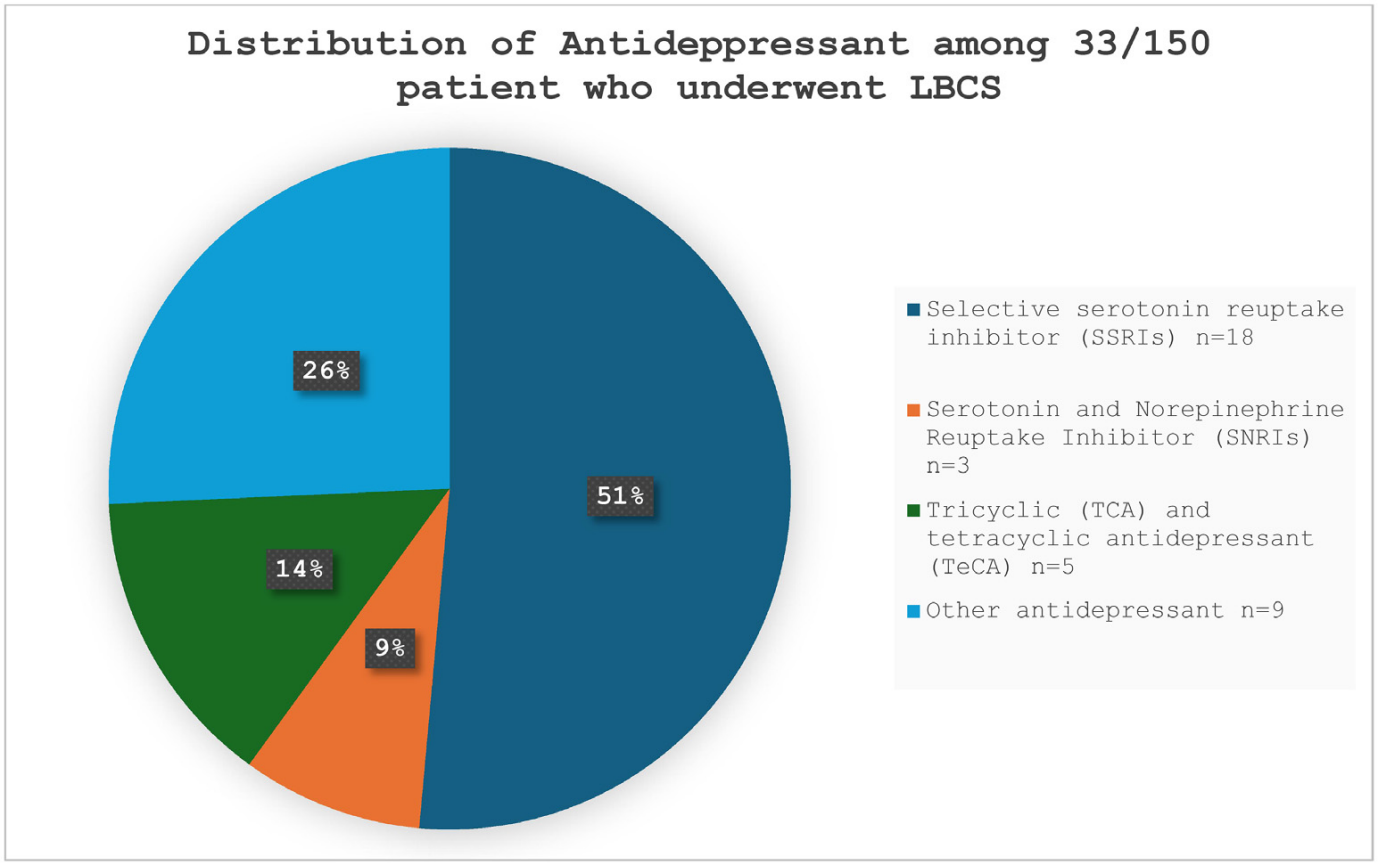
A total of 33/150 (22 %) patients used antidepressants. The most used antidepressant was selective serotonin reuptake inhibitor (SSRI).

In total, 33 patients (22 %) used antidepressants, and three patients used more than one simultaneously. The most commonly used antidepressant was an SSRI, taken by 18 patients (51 %) among those using antidepressants. Figure 1 illustrates the distribution of antidepressants across the different pharmacological groups.

Patients using antidepressants were notably older than non-users, with a mean age of 49.1 ± 10.1 years compared to 44.8 ± 11.2 years, *p *= 0.05. Among patients using antidepressants, 84.9 % had undergone BS, while only 15.1 % had lost weight through non-surgical methods. In comparison, 67.5 % of patients not using antidepressants had undergone BS, and 32.5 % had lost weight through a non-surgical method. The difference in weight loss methods between the groups approached statistical significance, *p *= 0.05. Antidepressant users had a higher BMI at the time of LBCS, 29.5 ± 5.2 kg/m^2^ vs. 27.8 ± 3.0 kg/m^2^, *p* = 0.02. and shorter hospital stay 3.3 ± 0.98 vs. 3.8 ± 1.3 days, *p* = 0.03, Table 1.

### Post-operative complications

**Figure 2:**
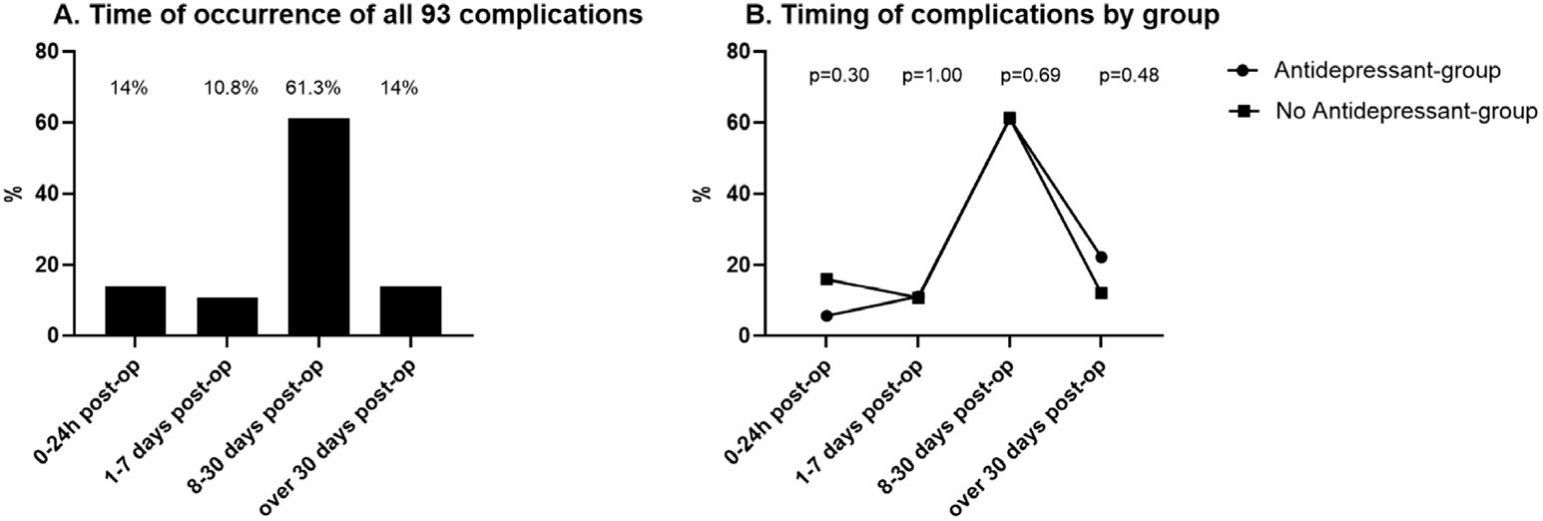
A–B. Time of complications occurrence since LBCS. A. Time of occurrence for all 93 complications. Most complications (61.3 %) were identified between 8 and 30 days post-operatively. B. The time of complication occurrence by group is based on the antidepressant. Immediate complications (0–24 h) occurred in **5.6 %** of patients using antidepressants and in 16 % of those not using antidepressants, without statistical difference (*p* = 0.30).

A total of 93 complications were recorded in 77 patients (51.3 %) following LBCS, Table 2. The most common complications were postoperative hematomas, observed in 19 patients (12.7 %), and seroma formation, observed in 18 patients (12 %). Wound healing complications of varying severity were observed in 39 patients (26 %). A total of 22 patients (14.7 %) required surgical intervention due to complications, most commonly for hematoma evacuation (14 out of 22 cases). Most complications (61.3 %) occurred between 8 and 30 days post-surgery. According to the Clavien-Dindo classification, the majority, 34 (44.2 %) of complications were classified as Grade II. Grade I complications occurred in 21 cases (27.3 %), and Grade III complications in 22 cases (28.6 %). No Grade IV or Grade V complications were observed in the cohort*.* Figure 2A illustrates the distribution of complications based on their time of onset following LBCS. Due to complications, reoperations were performed in 22 patients (14.7 %) from the total cohort.

**Table 2:** All 93 post-operation complications in 77/150 (51.3 %) patients after LBCS due to MWL. The *p*-value reflects the probability of the observed difference between two groups regarding the use of any antidepressant medication.

	All patients^b^	Antidepressant-group	Non antidepressant-group	*p*-value^a^
*n* (%)	150 (100)	33 (22)	117 (78)	
Patients with complications	77 (51.3)	15 (45.5)	62 (53.0)	0.44
All complications	93 (100)	18 (54.6)	75 (64.1)	0.32
Clavien-Dindo Classification				0.30
gradus I	21 (27.3)	2 (13.3)	19 (30.7)	
gradus II	34 (44.2)	9 (60)	25 (40.3)	
gradus III	22 (28.6)	4 (26.7)	18 (29)	
Hematoma	19 (12.7)	1 (3)	18 (15.4)	0.08
Seroma	18 (12)	5 (15.2)	13 (11.1)	0.55
**Wound healing complications**	39 (26)	6(18.2)	33 (28.2)	0.25
Local infection	10 (6.7)	2 (6.1)	8 (6.8)	0.29
Wounds dehisce	25 (16.7)	3 (9.1)	22 (18.8)	
Wound edge necrosis	1 (0.7)	0	1(0.9)	
Navel necrosis	4 (2.7)	1 (3)	3(2.69	
Unnormal pain	4 (2.7)	0	4 (3.4)	0.62
Deep tissue infection	11 (7.3)	5 (15.2)	6 (5.1)	0.07
Fat necrosis	1 (0.7)	1	0	
**Surgery due to complication**	22 (14.7)	4 (12.1)	18 (15.4)	0.79
Evacuation of hematoma	14 (9.3)	1 (3)	13 (11.1)	0.31
Revision of surgical wound	7 (4.7)	3 (9.1)	4 (3.4)	0.18
Excision of seroma capsule	1 (4.6)	0	1 (0.9)	

^a^Chi-square test.

^b^Parametric values are reported for the number of patients marked as *n* (%).

**Figure 3:**
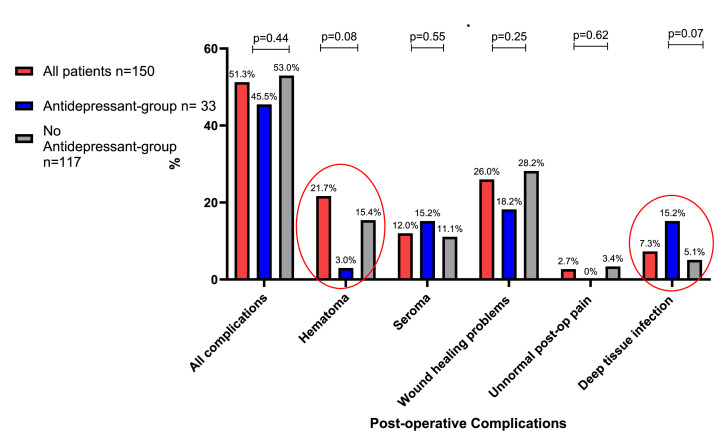
The percentages of postoperative complications within the groups. Patients using antidepressants had fewer postoperative hematomas compared to those who did not use antidepressant medication: 3 % vs. 15.4 %. However, deep tissue infections were considerably more common among antidepressant users: 15.2 % vs. 5.1 %. Nevertheless, the differences were not statistically significant.

The group using antidepressants had a slightly lower overall complication rate (45.5 % vs. 53 %), although the difference was not statistically significant (*p *= 0.44). Hematomas were observed in one patient (3 %) in the antidepressant group and in 18 patients (15.4 %) in the non-antidepressant group (*p *= 0.08). Deep tissue infections occurred in five patients (15.2 %) in the antidepressant group and six patients (5.2 %) in the non-antidepressant group (*p *= 0.07), Figure 3. The rate of reoperation due to complications also did not differ significantly between the groups (12.1 % vs. 15.4 %, *p* = 0.79).

### Subanalysis of the risk of complications

A multivariable logistic regression analysis, adjusted for BMI and age at the time of surgery, diabetes, hypertension, and smoking status, suggested that patients using any antidepressant had a reduced, although not statistically significant, risk of overall complications (aOR 0.73, 95 % CI: [0.32–1.68], *p* = 0.46), hematoma (aOR 0.13, 95 % CI: [0.03–1.58], *p *= 0.13), and wound healing problems (aOR 0.56, 95 % CI: [0.19–1.59], *p *= 0.27). In contrast, patients using antidepressants exhibited a significantly increased risk of deep tissue infection (aOR 4.36, 95 % CI: [1.08–17.71], *p *= 0.04), Table 3.

**Table 3:** The association between any antidepressants use, selective serotonin reuptake inhibitors (SSRI) and serotonin-norepinephrine reuptake inhibitors (SNRI) medication, and other antidepressant use and the risk of complications following lower body contouring surgery (LBCS).

Adjusted Odds Ratio (aOR) Estimates and Wald Confidence Intervals (Cl)															
Study variables	All complications			Hematoma			Wound healing complications			Seroma			Deep tissue infection		
	aOR	95 % Cl	*p*-value^a^	aOR	95 % Cl	*p*-value^a^	aOR	95 % Cl	*p*-value^a^	aOR	95 % Cl	*p*-value^a^	aOR	95 % Cl	*p*-value^a^
Using any Antidepressant	0.73	0.32–1.68	0.46	0.13	0.03–1.58	0.13	0.56	0.19–1.59	0.27	1.13	0.38–4.61	0.67	4.36	1.08–17.71	0.04^b^
Using SSRI+SNRI medication	0.66	0.25–1.78	0.42	0.36	0.04–2.94	0.34	0.46	0.12–1.72	0.25	1.20	0.29–5.01	0.81	3.75	0.80–13.23	0.09
Using Other antidepressant	1.05	0.31–3.61	0.94	1.00	0	0	0.37	0.06–2.17	0.27	4.98	1.03–24.10	˂0.05^b^	5.92	1.12–31.20	0.04^b^

^a^Multivariable Logistic regression analysis adjusted for BMI and age at the time of surgery, diabetes, hypertension and smoking status.

^b^Indicates a statistically significant finding. Statistical significance was set at *p* < 0.05.

A multivariable logistic regression analysis was performed to examine the association between any antidepressant use and the risk of complications requiring additional treatment following LBCS, adjusting for BMI and age at the time of surgery, length of hospital stay, diabetes, hypertension and smoking status. Antidepressant use was significantly associated with a delayed initiation of intravenous antibiotics (aOR 5.07, 95 % CI: [1.28–20.02], *p* = 0.02). Subgroup analysis revealed that both SSRI/SNRI users (aOR 4.88, 95 % CI: [1.16–20.53], *p* = 0.03) and patients using other types of antidepressants (aOR 7.81, 95 % CI: [1.62–37.61], *p* = 0.01) had a significantly higher risk of delayed antibiotic treatment, Table 4.

**Table 4:** The association between any antidepressant use, selective serotonin reuptake inhibitors (SSRI) and serotonin-norepinephrine reuptake inhibitors (SNRI) medication, and other antidepressant use with complications requiring additional treatment following lower body contouring surgery.

Adjusted Odds Ratio (aOR) Estimates and Wald Confidence Intervals (CI)									
Study variables	Surgery due complication			Delay intravenous antibiotic			Seroma punction at outpatient clinic		
	aOR	95 % Cl	*p*-value^a^	aOR	95 % Cl	*p*-value^a^	aOR	95 % Cl	*p*-value^a^
Antidepressant medication during BCS	1.17	0.33–4.21	0.81	5.07	1.28–20.02	0.02^b^	1.17	0.23–5.89	0.85
SSRI+SNRI Medication	2.21	0.58–8.38	0.25	4.88	1.16–20.53	0.03^b^	0.49	0.05–4.72	0.54
Other Antidepressant	0.00		1.000	7.81	1.62–37.61	0.01^b^	2.68	0.34–21.14	0.35

^a^Multivariable logistic regression analysis adjusted for BMI and age at the time of surgery and length of postoperative hospital stay, diabetes, hypertension and smoking status.

^b^Indicates a statistically significant finding. Statistical significance was set at *p* < 0.05.

## Discussion

In this retrospective single-center study, 22 % of patients who underwent LBCS following MWL were found to be using antidepressants, with SSRI/SNRIs being the most common. More than half of all patients (51.3 %) experienced some degree of postoperative complications following LBCS, consistent with previous studies.^9^^,^^10^ Patients using antidepressants had a significantly higher BMI at the time of LBCS, which may have partly contributed to their increased risk of postoperative complications. However, there was no significant difference in the overall complication rates between groups based on antidepressant use. Exploratory subgroup analysis suggested a potential association between antidepressant use and specific complications, such as deep tissue infections and subsequent rehospitalization for intravenous antibiotics. These findings should, however, be interpreted with caution due to the limited sample size and the non-pre-specified nature of the analysis. Given the high complication rate associated with LBCS, operating surgeons should be aware of these risks and consider the potential impact of antidepressants on postoperative recovery. Rather than altering psychiatric treatment, a multidisciplinary preoperative assessment involving both surgical and mental health professionals may help to optimize patient outcomes and guide postoperative care planning

Weight loss has a positive effect on depression and improves the overall QoL.^21^ Guest et al. found that the prevalence of depression was 42 % before MWL, which decreased to 32 % following MWL, regardless of the method of weight loss (*p* ˂ 0.001).^22^ In this study, as many as 22 % of patients used antidepressant medication following MWL. Although we were unable to estimate the prevalence of clinical depression based solely on antidepressant use, the fact that such use was markedly more common than in the general population, where the prevalence of depression is estimated at approximately 5–7 % among adults,^23^^,^^24^ suggests that clinically significant depressive and anxiety symptoms were more prevalent among patients undergoing LBCS.^25^ It should also be noted that this study did not examine antidepressant use prior to weight loss or prior to BS, as some procedures were performed outside the study center, and more detailed patient records were unavailable. Therefore, we were unable to assess whether antidepressant use decreased following massive weight loss.

Excess skin folds following MWL, along with various psychosocial and health-related symptoms, can impair the patient's QoL and increase the risk of depression.^3^ This may partially explain the increased use of antidepressants. Surgical intervention, specifically LBCS, remains the only effective treatment for removing excess skin folds. Postoperative complications after LBCS are relatively common. Our findings support this, with an overall complication rate of 51.3 % observed in the present study*.* Some minor complications may have gone undocumented if treated at local healthcare centers; therefore, the true complication rate may be slightly higher. Nevertheless, all patients attended a routine follow-up visit approximately 4–6 weeks after surgery, during which such externally managed issues were typically reported by the patient and documented in the medical records and were therefore likely captured in the present analysis. There were no statistically significant differences in the prevalence of overall complications between antidepressant users and non-users (45.5 % vs.53.0 %). In this study, no cases of severe complications, such as venous thrombosis, pulmonary embolism, or mortality, were observed.

In this study, deep tissue infections were more prevalent among patients using antidepressants than among those who did not use them: 15.2 % vs. 5.1 %. A more detailed analysis revealed that patients using antidepressants had nearly a five-fold increased risk of rehospitalization due to deep infection and the need for intravenous antibiotic treatment. This finding is notable, despite postoperative antibiotics being prescribed to over 70 % of all patients. It can be speculated whether patients discontinued their antibiotic treatment after discharge, leading to deep infections, or whether patients with depression failed to adhere to wound care instructions, thereby increasing the risk of infection. Similar findings were reported by Doering et al., who examined patients undergoing coronary artery bypass surgery and their depressive symptoms. They found that patients with more depressive symptoms were significantly more likely to require rehospitalization for infection-related complications than those experiencing lower levels of psychological distress.^26^

Patients’ psychological health, including stress, anxiety, and depression, may increase the incidence of postoperative wound healing problems.^27^, ^28^, ^29^ This may be due to several factors, including altered immune responses and elevated levels of inflammation.^30^ Typically, these complications can be managed with local treatment, without the need for prolonged hospitalization or revision surgery. In this study, wound healing issues of varying severity, including local wound infection, wound dehiscence, wound edge necrosis, and navel necrosis, were observed in 26 % of patients. No differences in the prevalence of postoperative wound healing complications were found between antidepressant users and non-users, even though patients on antidepressants had a significantly higher BMI at the time of surgery. Elevated BMI has been identified in several previous studies as a risk factor for impaired wound healing, particularly when combined with recent weight loss or weight reduction methods such as bariatric surgery.^10^^,^^31^^,^^32^ Nutritional status, smoking, and comorbidities such as diabetes are also recognized risk factors for postoperative complications.^9^^,^^31^ However, this study did not assess patients' nutritional status at the time of surgery, but comorbidities such as diabetes and hypertension, as well as smoking status, were taken into account in the analysis. All patients in our study cohort received perioperative antibiotic prophylaxis. Data regarding the effectiveness of preoperative antibiotics in preventing postoperative infections following BCS are contradictory.^33^^,^^34^ Swedenhammer et al. demonstrated that a single dose of preoperative antibiotic prophylaxis appears to reduce the risk of wound infection.^34^

Serotonergic antidepressants are thought to increase intraoperative and postoperative bleeding, as serotonin affects platelet function and plays an important role in platelet aggregation, thereby impairing blood clotting.^35^^,^^36^ However, previous studies suggest that this assumption remains inconclusive.^19^^,^^37^ *Postoperative hematoma remains one of the most significant complications following LBCS as it may threaten patient safety, delay wound healing and contribute to skin necrosis.* In our study, the overall incidence of postoperative hematoma was 12.7 %, which aligns with findings from previous studies.^22^^,^^31^ *At our institution, several preventive measures are routinely implemented to minimize this risk. Preoperatively, patients are optimized in terms of comorbidities and anticoagulant therapy. Intraoperatively, meticulous hemostasis, gentle tissue handling, and the routine use of closed-suction drains are essential. Postoperatively, compression dressings, close monitoring, and prompt reoperation in suspected cases are crucial. Collectively, these measures may reduce the incidence and severity of hematomas.*

In this study the incidence of postoperative hematomas was lower among patients using antidepressants compared to those who were not, 3 % vs. 15.4 %. Our study showed that the use of SSRI and SNRI medications, as well as antidepressants in general, was associated with a reduced risk of postoperative hematoma. Several possible explanations may account for this unexpected finding. One potential explanation is the relatively small sample size of antidepressant users in our study, which may have introduced a chance effect. Additionally, the heterogeneity of SSRI and SNRI medications, which vary in their effects on platelet function, may also contribute. However, this finding may help explain why patients using antidepressants had a significantly shorter hospital stay after LBCS compared to those not using these medications. This is a noteworthy finding, as previous studies have generally shown that patients with psychiatric disorders tend to have slower recovery and more extended hospital stays.^38^

This study has several strengths. Firstly, it included a well-defined cohort of patients who underwent LBCS following MWL. The main strength of the study lies in the ability to conduct detailed individual chart reviews, enabling the extraction and verification of high-quality clinical data. Furthermore, the study focused on a relatively underexplored area—the association between antidepressant use and specific postoperative complications, providing preliminary insights into this patient population. However, as a retrospective observational study, the analyses are exploratory and descriptive in nature, and no causal inferences can be drawn.

This study has some limitations. As a retrospective study, it is subject to inherent biases, particularly documentation bias, which can be especially problematic when identifying complications that were either not clearly recorded or were managed outside the study institution. Additionally, as a single-center study, the generalizability of the findings may be limited in settings with different patient populations or clinical practices*.* The subgroup analyses comparing SSRI and non-SSRI medications should be interpreted as hypothesis-generating, as they are based on a relatively small sample size, which limits the robustness of the conclusions and warrants cautious interpretation. One limitation is the lack of information regarding the indications for the use of antidepressants. For example, antidepressants may be prescribed for anxiety disorders as well as depression, and we can only speculate on the specific psychological distress of the patients. In addition, antidepressant use was defined solely based on prescription records, without confirmation of actual intake or duration of use. This may have resulted in exposure misclassification, particularly in psychiatric populations where non-adherence is common. Future multicenter prospective studies should incorporate more accurate measures of adherence—such as pharmacy refill data, medication possession ratios, or patient self-reports—and include validated patient-reported outcome (PRO) instruments to better assess the relationship between antidepressant use and surgical outcomes.

## Conclusion

This study highlights the high prevalence of antidepressant use among patients undergoing LBCS following MWL, as well as its potential impact on post-operative complications. Antidepressant use may be associated with an increased risk of deep infections after LBCS. Given the high complication rates, a multidisciplinary approach is essential for optimizing patient care. Surgeons should be aware of the potential risks associated with antidepressant use and consider strategies to minimize infections and enhance post-discharge care. Further research is needed to refine preoperative assessments and improve postsurgical outcomes in this patient population.

## Funding

None.

## Ethical approval

Not required.

## Declaration of competing interest

None declared.

## References

[1] Gusenoff J.A. (2019). Body contouring after massive weight loss. Clin Plast Surg.

[2] Toma T., Harling L., Athanasiou T., Darzi A., Ashrafian H. (2018). Does body contouring after bariatric weight loss enhance quality of life? A systematic review of QoL studies. Obes Surg.

[3] Staalesen T., Olsen M.F., Elander A. (2013). Experience of excess skin and desire for body contouring surgery in post-bariatric patients. Obes Surg.

[4] de Zwaan M., Georgiadou E., Stroh C.E. (2014). Body image and quality of life in patients with and without body contouring surgery following bariatric surgery: a comparison of pre- and post-surgery groups. Front Psychol.

[5] Biorserud C., Olbers T., Olsen M.F. (2011). Patients’ experience of surplus skin after laparoscopic gastric bypass. Obes Surg.

[6] Monpellier V.M., Antoniou E.E., Mulkens S., Janssen I.M.C., van der Molen A.B.M., Jansen A.T.M. (2018). Body image dissatisfaction and depression in postbariatric patients is associated with less weight loss and a desire for body contouring surgery. Surg Obes Relat Dis.

[7] Sierżantowicz R., Ładny J.R., Lewko J. (2022). Quality of life after bariatric surgery—a systematic review. Int J Environ Res Public Health.

[8] Pajula S., Gissler M., Jyränki J., Tukiainen E., Koljonen V. (2020). Actualized lower body contouring surgery after bariatric surgery—a nationwide register-based study. J Plast Surg Hand Surg.

[9] Botero A.G., Wenninger M.G., Loaiza D.F. (2017). Complications after body contouring surgery in postbariatric patients. Ann Plast Surg.

[10] Pajula S., Jyranki J., Tukiainen E., Koljonen V. (2019). Complications after lower body contouring surgery due to massive weight loss unaffected by weight loss method. J Plast Reconstr Aesthet Surg.

[11] Parvizi D., Friedl H., Wurzer P. (2015). A multiple regression analysis of postoperative complications after body-contouring surgery: a retrospective analysis of 205 patients : regression analysis of complications. Obes Surg.

[12] De Paep K., Van Campenhout I., Van Cauwenberge S., Dillemans B. (2021). Post-bariatric abdominoplasty: identification of risk factors for complications. Obes Surg.

[13] Marouf A., Mortada H. (2021). Complications of body contouring surgery in postbariatric patients: a systematic review and meta-analysis. Aesthetic Plast Surg.

[14] Wes A.M., Wink J.D., Kovach S.J., Fischer J.P. (2015). Venous thromboembolism in body contouring: an analysis of 17,774 patients from the National Surgical Quality Improvement databases. Plast Reconstr Surg.

[15] Ghoneim M.M., O’Hara M.W. (2016). Depression and postoperative complications: an overview. BMC Surg.

[16] Gouin J.P., Kiecolt-Glaser J.K. (2012). The impact of psychological stress on wound healing: methods and mechanisms. Crit Care Nurs Clin North Am.

[17] Geoffrion R., Koenig N.A., Zheng M. (2021). Preoperative depression and anxiety impact on inpatient surgery outcomes: a prospective cohort study. Ann Surg.

[18] Roose S.P., Rutherford B.R. (2016). Selective serotonin reuptake inhibitors and operative bleeding risk: a review of the literature. J Clin Psychopharmacol.

[19] Duncan A., Stewart K., Dow T., Williams J. (2024). Incidence of hematoma following breast reduction in patients taking selective serotonin reuptake inhibitor or serotonin-norepinephrine reuptake inhibitor: a retrospective review. Cureus.

[20] Clavien P.A., Barkun J., de Oliveira M.L. (2009). The Clavien-Dindo classification of surgical complications: five-year experience. Ann Surg.

[21] Mazer L.M., Azagury D.E., Morton J.M. (2017). Quality of life after bariatric surgery. Curr Obes Rep.

[22] Guest R.A., Bourne D.A., Chow I., Gusenoff J.A., Peter Rubin J. (2019). The impact of massive weight loss on psychological comorbidities: a large, retrospective database review. Aesthetic Plast Surg.

[23] Markkula N., Suvisaari J., Saarni S.I. (2015). Prevalence and correlates of major depressive disorder and dysthymia in an 11-year follow-up—results from the Finnish Health 2011 Survey. J Affect Disord.

[24] Pirkola S.P., Isometsä E., Suvisaari J. (2005). DSM-IV mood-, anxiety- and alcohol use disorders and their comorbidity in the Finnish general population–results from the Health 2000 Study. Soc Psychiatry Psychiatr Epidemiol.

[25] Bojanić I. (2024). Use of antidepressant and anxiolytic drugs in Scandinavian countries between 2006 and 2021 : a prescription database study. Depress Anxiety.

[26] Doering L.V., Moser D.K., Lemankiewicz W., Luper C., Khan S. (2005). Depression, healing, and recovery from coronary artery bypass surgery. Am J Crit Care.

[27] Ghoneim M.M., O’Hara M.W. (2016). Depression and postoperative complications: an overview. BMC Surg.

[28] Gouin J.P., Kiecolt-Glaser J.K. (2012). The impact of psychological stress on wound healing: methods and mechanisms. Crit Care Nurs Clin North Am.

[29] Geoffrion R., Koenig N.A., Zheng M. (2021). Preoperative Depression and anxiety impact on inpatient surgery outcomes: a prospective cohort study. Ann Surg.

[30] Bufalino C., Hepgul N., Aguglia E., Pariante C.M. (2013). The role of immune genes in the association between depression and inflammation: a review of recent clinical studies. Brain Behav Immun.

[31] Hunecke P., Toll M., Mann O., Izbicki J.R., Blessmann M., Grupp K. (2019). Clinical outcome of patients undergoing abdominoplasty after massive weight loss. Surg Obes Relat Dis.

[32] Romano L., Zoccali G., Orsini G., Giuliani M. (2019). Reducing complications in post-bariatric plastic surgery: our experience and literature review. Acta Biomed.

[33] Ariyan S., Martin J., Lal A. (2015). Antibiotic prophylaxis for preventing surgical-site infection in plastic surgery: an evidence-based consensus conference statement from the American Association of Plastic Surgeons. Plast Reconstr Surg.

[34] Swedenhammar E., Stark B., Hållstrand A.H., Ehrström M., Gahm J. (2018). Surgical training and standardised management guidelines improved the 30-day complication rate after abdominoplasty for massive weight loss. World J Surg.

[35] Auerbach A.D., Vittinghoff E., Maselli J., Pekow P.S., Young J.Q., Lindenauer P.K. (2013). Perioperative use of selective serotonin reuptake inhibitors and risks for adverse outcomes of surgery. JAMA Intern Med.

[36] Teo I., Song C.T. (2015). Assessing the risks associated with antidepressant use in plastic surgery: a systematic review. Plast reconstruct surg.

[37] Gärtner R., Cronin-Fenton D., Hundborg H.H. (2010). Use of selective serotonin reuptake inhibitors and risk of re-operation due to post-surgical bleeding in breast cancer patients: a Danish population-based cohort study. BMC Surg.

[38] McBride K.E., Solomon M.J., Bannon P.G., Glozier N., Steffens D. (2021). Surgical outcomes for people with serious mental illness are poorer than for other patients: a systematic review and meta-analysis. Med J Australia.

